# Neurologic Abnormalities in HIV-1 Infected Children in the Era of Combination Antiretroviral Therapy

**DOI:** 10.1371/journal.pone.0064398

**Published:** 2013-05-15

**Authors:** Lotus A. van Arnhem, Madeleine J. Bunders, Henriette J. Scherpbier, Charles B. L. M. Majoie, Liesbeth Reneman, Olivier Frinking, Bwee Tien Poll-The, Taco W. Kuijpers, Dasja Pajkrt

**Affiliations:** 1 Department of Pediatric Hematology, Immunology and Infectious Diseases, Emma Children’s Hospital, Academic Medical Centre (AMC), Amsterdam, The Netherlands; 2 Department of Radiology, AMC, Amsterdam, The Netherlands; 3 Department of Pediatric Neurology, Emma Children’s Hospital, AMC, Amsterdam, The Netherlands; University of Nebraska Medical Center, United States of America

## Abstract

**Background:**

Pediatric HIV-1 infection is associated with neurologic abnormalities. In recent years, the neurological outcome of HIV-1 infected children has substantially improved with combination antiretroviral therapy (cART). However, data regarding the long-term effect of cART and neurologic outcome are limited.

**Methods:**

In the Pediatric Amsterdam Cohort on HIV-1 study, 59 perinatally HIV-1 infected children were evaluated from 1992–2010. All children underwent neurological examination and neuro-imaging studies, including CT-scan and/or MRI imaging. Fisher exact and Kruskal-Wallis tests were used to compare clinical deviations of neuro-imaging studies with HIV-1 related parameters, including CD4^+^ T cell count, HIV-1 viral load in blood and cerebrospinal fluid (CSF), and duration of cART as well as neurological examination.

**Results:**

Abnormal neurologic examinations in these HIV-1 infected children included language impairment (22%), abnormal muscle tone (hyper/hypotonia) (14%) and delay in reaching developmental milestones (12%). Ventricular enlargement and sulcal widening (29%) and white matter lesions (38%) were prominent findings. White matter lesions were positively correlated with HIV-1 viral load levels. In a small follow-up sub study white matter lesions did not improve while children with ventricular enlargement and sulcal widening showed improvements whilst being treated with cART.

**Conclusions:**

In the current era of cART HIV-1 infected children still frequently show neurological impairments together with abnormal neuro-imaging.

## Introduction

Pediatric HIV-1 infection continues to be a worldwide challenge. Although the number of HIV-1 infected children is slowly decreasing, in 2009 still 370.000 children were newly HIV-1 infected via mother-to-child-transmission (MTCT) [1].

HIV-1 associated illnesses affect multiple organs and being a neurotrophic virus, HIV-1 can severely affect the central nervous system (CNS). In the pretreatment era, classic pediatric HIV-1 encephalopathy (as characterized by impaired brain growth, motor deficits and developmental delay) was the most prevalent neuro-disability reported [2]–[5]. In addition, HIV-1 infected children can present with more variable neurologic symptoms, including seizures, headaches, and behavioral changes [6], [7]. Neuro-imaging of (perinatally) HIV-1 infected children shows substantial abnormalities of both the white and grey matter, such as white matter lesions and calcifications [8]–[12].

Treatment of HIV-1 infected children with combination antiretroviral therapy (cART) has substantially improved the clinical outcome of HIV- 1 infected children and resulted in a decrease of plasma and cerebrospinal fluid (CSF) HIV-1 RNA viral load (HIV VL). The incidence of HIV-1 encephalopathy has decreased significantly in the cART era [13]–[15]. More recently, early ART intervention proved effective in improving neurodevelopmental outcomes in infants [16]. Nevertheless, data from adult infected individuals suggest that the CNS can serve as a reservoir for HIV-1 and viral replication and immune activation can take place whilst being treated with cART, potentially associated with on-going neurological damage [17]. Up to date there is a lack of data regarding the follow-up of neurological impairments and neuro-imaging abnormalities in HIV-1 infected children receiving long-term cART. In this study, we evaluated neurologic functioning and neuro-imaging findings in perinatally HIV-1 infected children who were treated with cART and associated HIV-1 parameters.

## Methods

### Ethics Statement

The ethics committee of the Academic Medical Centre, Amsterdam, approved the study. Written informed consent was obtained from all study participants and the study was conducted according to guidelines of the declaration of Helsinki.

### Subjects

Here, we describe a single-center observational cohort study. From January 1992 until May 2010 59 HIV-1 infected children treated at the Emma Children’s Hospital Academic Medical Centre in Amsterdam, the Netherlands were included in the study. These children also participated in the Pediatric Amsterdam Cohort on HIV-1 (PEACH) study, that evaluated long-term effectiveness and safety of cART in HIV-1 infected children [18]. All included children were infected via MTCT of HIV-1. None of the mothers used illicit drugs during pregnancy. Antiretroviral treatment regimes of the mothers of included patients were not documented.

### Antiretroviral Treatment

Before 1996, all included children received zidovudine, monotherapy or in combination with either lamivudine or didanosine, as part of standard patient care. In 1996 cART was introduced in the Netherlands and consisted of two nucleoside reverse transcriptase inhibitors (NRTIs) in combination with either a protease inhibitor (PI) or a non-nucleoside reverse transcriptase inhibitor (NNRTI). The two combinations of cART most frequently used in this study were either a combination treatment with nelfinavir, lamivudine and stavudine (mostly used from 1996 until 2002) or a combination treatment with efavirenz, abacavir, didanosine and lamivudine (mostly used from 2002 until 2005) [19].

### Drug Resistance Mutations

Drug resistance mutation analysis was performed in the first available blood samples from all patients prior to start cART and were interpreted according to the 2013 IAS-USA drug resistance list [20]. HIV-1 sequence analysis was performed with the Viroseq HIV-1 genotyping kit (version 2; Abbott Laboratories, Abbott Park, IL) as reported previously [21]. Alternative switches of antiretroviral treatment regimes were based on newly acquired drug resistance mutations to cART components.

### Neuro-imaging and Neurological Examination

Neuro-imaging was performed by computed tomography (CT) from 1992 until 2001 and by magnetic resonance imaging (MRI) after 2001. CT scans were performed with 5 mm slice thickness without contrast enhancement. MRI was performed on 1.5 T scanners using axial T1- and T2-weighted spin echo and FLAIR sequences. The neuro-imaging scans were evaluated by two independent radiologists and scored for presence or absence of intracerebral calcifications, ventricular enlargement, sulcal widening and number of white matter lesions using a standardized scoring list. Baseline is defined as the time of the first neuro-imaging of a child. In a sub-study, we randomly included 19 children for a follow-up neuro-imaging examination to evaluate alterations in neuro-imaging over time.

A standard neurological physical examination was performed yearly at the outpatient clinic by the treating pediatrician and pediatric neurologist and included evaluation of cranial nerves, motor function, sensory function, reflexes, and coordination. Developmental delay and language impairment was documented are present or absent using standardized age appropriate developmental milestone charts as part of standard patient care. The above described neurological outcome parameters were described as a cumulative variable of all neurological examinations up to baseline, which was the time of first neuro-imaging.

Ethnicity was defined by the ethnicity of the mother and father and described as black, caucasian, mixed black or other. Progression of disease was determined using Centers for Disease Control and Prevention (CDC) classification. Children with C classification were defined as progressed to AIDS. HIV-1 positivity was determined by detection of HIV-1 antibodies after 18 months of age or by a positive RNA PCR in the first 18 months of life. Until 2001 plasma and cerebrospinal HIV VL was quantified using the NucliSens assay (Biomerieux, Boxtel, the Netherlands). From 2001 until 2007 the Versant HIV-1 RNA 3.0 Assay (bDNA, Siemens Bayer Diagnostics, Tarrytown, NY) was used and after 2007 the Abbott Real Time HIV-1 assay (Abbott Molecular Inc., Des Plaines, IL). Blood samples for HIV VL and CD4^+^ T cell percentage were collected during regular outpatient clinic visits. At the time of the first neuro-imaging examination, CSF analysis was performed for HIV VL detection in 29 children, randomly assigned.

### Statistical Analyses

Data entry and management were carried out using Microsoft Office Access, 2007 (Microsoft Corp, Redmond, WA, USA). Statistical analyses were carried out using StataIC 10, 2009 (StataCorp, College Station, TX, USA). The following variables were included in the analyses: nadir CD4^+^ T cell percentage, CD4^+^ T cell percentage at time of neuro-imaging, peak HIV VL and HIV VL at time of neuro-imaging as well as CDC classification at time of neuro-imaging.

Results are summarized using medians with inter-quartile ranges (IQR) and proportions. Comparisons between outcome variables such as number of white matter lesions or ventricular enlargement and/or sulcal widening were made by using Fisher’s exact test for categorical data and the Kruskal-Wallis test for continuous data. In the statistical analyses we combined ventricular enlargement and sulcal widening because of frequent simultaneous occurrence. The level of critical significance was assigned at *p*<0.05.

## Results

### Baseline Sub Study

At baseline which is at time of first neuro-imaging, characteristics of 59 HIV-1 infected children were evaluated as depicted in Table 1. Most children were black (68%). CDC classification B or C was diagnosed in 15 and 25 children respectively. The majority of children were moderately immune compromised at initiation of ART, with a median CD4^+^ T cell nadir of 20% (IQR: 9% to 34%). The median age at baseline was 4.9 years (IQR: 1.5 to 8.9 years). The majority of children (n = 31) received cART at baseline, and 14 children had undetectable HIV VL in blood at baseline neuro-imaging. The median age at initiation of ART was 2.6 years (IQR: 0.8 to 5.9 years). At baseline, 12 children had been exposed to a PI-based regimen (7 patients to nelfinavir, 4 patients to lopinavir/ritonavir and 1 patient to indinavir/ritonavir), 9 children to a NNRTI-based regime (6 children to efavirenz and 3 patients to nevirapine), 10 children had been exposed to PI and NNRTI based regimens (various combinations of nelfinavir or lopinavir/ritonavir and/or nevirapine or efavirenz) (see Table 1).

**Table 1:** Characteristics of the 59 children at baseline, at time of first neuro-imaging.

Characteristics	
Sex, N (%)	
Male	28 (47)
Female	31 (53)
Ethnicity, N (%)	
Black	40 (68)
Caucasian	6 (10)
Black/caucasian	7 (12)
Other^1^	6 (10)
Date of birth, N (%)	
Before 1996	19 (32)
Between 1996–2003	34 (58)
After 2003	6 (10)
CDC-classification, N (%)	
N	5 (9)
A	13 (22)
B	15 (25)
C	25 (42)
Unknown	1 (2)
CD4^+^ T cell % nadir	20
Median (IQR)	(9–34)
CD4^+^ T cell % at baseline	29
Median (IQR)	(19–36)
Lost to follow-up, N (%)	2 (3)
ART experience, N (%)	
No therapy	5 (8)
Mono or dual therapy	2 (3)
PI-based cART	12 (20)
NNRTI-based cART	9 (15)
Both PI- and NNRTI-based cART	10 (17)
Duration of ART experience	1.6
Median years (IQR)	(0.3–5.8)
Age at start ART	2.6
Median years (IQR)	(0.8–5.9)
HIV VL <400 copies/ml, N (%)	14 (24)
HIV VL at start ART	5.3
Median log copies/ml (IQR)	(4.4–6.0)

ART, antiretroviral therapy; PI, protease inhibitor; NNRTI, non-nucleoside reverse transcriptase inhibitor; VL, viral load;

1 Other included Asian ethnicity or unknown.

Drug resistance mutation analyses from first available plasma samples (n = 53) could not be performed in 4 patients because of undetectable HIV VL.

There were no NNRTI or PI resistance mutations identified. In 2 patients major gene mutations associated with NRTI resistance were detected. One patient had mutations that confer high-resistance to multi-NRTIs (41 L, 44 D, 210 W and 215 Y) and one patient had a mutation that confers resistance to NRTI (184 V) at baseline. The first patient was pre-treated with zidovudine prior to start of cART. Both patients were optimally HIV suppressed with their initial cART that consisted of stavudine, didanosine and nelfinavir.

In 27 patients, the following minor gene mutations were detected: 69 N (NRTI), 10 I, 10 V, 13 V, 16 E, 20 I, 20 R, 36 I, 63 P, 64 L/V, 69 K, 77 I, 82 I, 93 L (atazanavir, ritonavir, saquinavir, indinavir, lopinavir or nelfinavir) and 138A (etravirine) in first available plasma samples prior to start cART. These mutations are not associated with drug resistance [20].

CSF analysis was performed in 29 children at time of first neuro-imaging. The median CSF HIV VL was 2.7 log copies/ml (IQR: 2.4 to 3.8 log copies/ml). There were no children with a detectable HIV VL in CSF while they simultaneously had an undetectable HIV VL in blood.

Neurological examination was abnormal in 17 of 59 children (29%) at time of first neuro-imaging (table 2). These abnormalities included hyperreflexia (10%), abnormal muscle tone, either hypertonia or hypotonia in 14%, spasticity and developmental delay in respectively 7% and 12% of the children. Language impairment was found in 13 children (22%). CT scans were performed in 20 children (prior to 2001) while MRI imaging was performed in 39 children (table 2) as first neuro-imaging test. Ventricular enlargement and/or sulcal widening was detected in 17 of 59 children (29%). White matter lesions (that can only be detected accurately by MRI imaging) occurred in 15 of 39 children (38%). Calcifications in basal ganglia were detected in one child.

**Table 2:** Neurologic examination and Neuro-imaging results, N = 59.

Findings* N (%)	
**Neurologic examination**	
Hyperreflexia	6 (10)
Spasticity	5 (8)
Hypertonia/hypotonia	8 (14)
Developmental delay	7 (12)
Language impairment	13 (22)
Total	17* (29)
**Neuro-imaging (CT, n = 20, MRI, n = 39)**	
Calcification	1 (2)
Widening	17 (2)
Only ventricular enlargement	6 (10)
Only sulcal widening	6 (10)
Ventricular enlargement and sulcal widening	5 (8)
White matter lesions	15 (38)
<3 lesions	7 (18)
>3 lesions	2 (5)
Diffuse	6 (15)

* Various findings could occur simultaneously.

HIV-1 related parameters of children with either ventricular enlargement and sulcal widening or white matter lesions were compared to those of children without abnormalities on neurological examination and neuro-imaging. Results are summarized in table 3. The incidence of ventricular enlargement and sulcal widening was higher in children diagnosed with AIDS, but did not reach statistical significance (*p* = 0.07). Other HIV-1 related parameters were not significantly associated with ventricular enlargement and sulcal widening. White matter lesions were significantly associated with higher peak HIV VL in both blood and CSF (*p* = 0.04). Additionally, there was a trend towards the presence of white matter lesions in children with a CD4^+^ T cell % of less than 15% (*p* = 0.06).There was no significant association between ventricular enlargement and/or sulcal widening and CD4^+^ T cell %.

**Table 3:** Neuro-imaging abnormalities and associations with HIV-1-related parameters.

	ventricular enlargement and/or sulcal widening (CT and MRI, N = 59)		white matter lesions (MRI,N = 39)	
Variable	Present (N = 17)	*p*-value	Present (N = 15)	*p*-value
Age	3.3	0.136	5.3	0.419
Median years (IQR)	(IQR 1.3–5.2)		(IQR 1.8–9.5)	
AIDS N (%)				
Yes	24 (42%)	0.066	9 (56%)	0.208
No	35 (20%)		30 (33%)	
CD4^+^ T cell % at neuro-imaging N (%)				
<15%	10 (40%)	0.308	7 (71%)	0.062
>15%	49 (27%)		32 (31%)	
CD4^+^ T cell % nadir				
N (%)				
<15%	20 (40%)	0.146	12 (58%)	0.090
>15%	39 (23%)		27 (30%)	
HIV VL peak	5.5	0.284	5.5	0.035
Median log copies/ml (IQR)	(IQR 4.4–6.2)		(IQR 4.7–6.0)	
HIV VL in CSF N (%)				
<500	13 (38%)	0.223	10 (20%)	0.035
>500	16 (19%)		10 (70%)	
Date of birth N (%)				
<1996	19 (21%)	0.279	8 (50%)	0.360
1996–2003	34 (29%)	0.571	25 (40%)	0.534
>2003	6 (50%)	0.225	6 (17%)	0.237
Duration of ART	0.9	0.828	3.5	0.702
Median years (IQR)	(IQR 0.04–2.9)		(IQR 1.1–6.5)	
Duration PI- regimen	0.08	0.481	3.7	0.786
Median years(IQR)	(IQR 0.02–2.8)		(IQR 1.3–4.9)	
Duration NNRTI-regimen	1.1	0.586	1.1	0.877
Median years (IQR)	(IQR 0.4–2.9)		(IQR 0.1–1.3)	

Continuous data are described as median and interquartile ranges. Statistical comparisons were made by using Fisher’s exact test for categorical data and the Kruskal-Wallis test for continuous data. AIDS, acquired immunodeficiency syndrome; HIV VL, HIV viral load; CSF, cerebrospinal fluid; cART, combination antiretroviral therapy; PI, protease inhibitor; NNRTI, non-nucleoside reverse transcriptase inhibitor; CT, computed tomography; MRI, magnetic resonance imaging.

Duration of cART prior to neuro-imaging did not impact the presence of ventricular enlargement and sulcal widening or white matter lesions. Even though the median duration of cART was shorter in children with ventricular enlargement and sulcal widening, these differences did not reach statistical significance. We replicated this finding in a second analysis including all children with a MRI during the whole study period, including additional 14 MRIs. There was no association with white matter lesions and duration of cART prior to MRI imaging (*p* = 0.93) (data not shown). We investigated if presence of neurological abnormalities at physical examination was associated with presence of abnormalities as detected by neuro-imaging. Ventricular enlargement and sulcal widening were associated with developmental delay and language impairment (*p* = 0.017) (Table 4). The analyses of white matter lesions did not show significant associations with neurological abnormalities. One child had had a toxoplasmosis infection and one was diagnosed with a congenital CMV infection, both conditions are related to abnormalities at neuro-imaging.

**Table 4:** Associations between ventricular enlargement and/or sulcal widening and white matter lesions and neurologic examination HIV-1 infected children.

	ventricular enlargement and/or sulcal widening (CT+MRI)			white matter lesions (MRI)		
Variable	N = 59	Present(N = 17)	*p*-value	N = 59	Present(N = 15)	*p*-value
Hyperreflexia N (%)						
Yes	6	4 (67%)	0.052	1	1 (100%)	0.385
No	53	13 (25%)		38	14 (37%)	
Spasticity N (%)						
Yes	5	3 (60%)	0.138	1	1 (100%)	0.385
No	54	14 (26%)		38	14 (37%)	
Hypertonia/hypotonia N (%)						
Yes	8	4 (50%)	0.157	2	1 (50%)	0.628
No	51	13 (25%)		37	14 (38%)	
Developmental delay N (%)						
Yes	7	5 (71%)	0.017	4	2 (50%)	0.502
No	52	12 (23%)		35	13 (37%)	
Language impairment N (%)						
Yes	12	7 (58%)	0.017	6	1 (17%)	0.237
No	47	10 (21%)		33	14 (42%)	

Statistical comparisons were made by using Fisher’s exact test for categorical data.

CT, computed tomography; MRI, magnetic resonance imaging.

### Longitudinal Sub Study

Of 59 HIV-1 infected children, only 19 children were randomly included in a follow up study. Five children had a second MRI. The other 14 children had a CT scan at baseline and a follow up MRI. The median duration between CT and MRI or between 2 MRIs was 5.9 years (IQR 1.7–9.2) for ventricular and/or sulcal widening and 7.5 years (IQR 5.4–8.6) for white matter lesions respectively. The median duration of cART prior to second neuro-imaging was 6.5 years (IQR 4.4–7.8) and 6.1 years (IQR 5.7–10.2) respectively. Nine children had ventricular enlargement and/or sulcal widening at baseline and in 4 children this finding had resolved on the follow up MRI. White matter lesions detected in 4 of 5 children that underwent a second MRI remained stable over time. Ventricular enlargement and/or sulcal widening as measured with CT and MRI improved in 5 children. Statistical analyses to identify associations between ventricular enlargement and/or sulcal widening ventricular widening or white matter lesions with CD4^+^ T cell %, HIV-1 VL and cART did not differ from baseline analyses (data not shown).

## Discussion

Our study describes the neurologic abnormalities detected in a retrospective cohort of perinatally HIV-1 infected children enrolled over a period of 18 years, from 1992 to 2010. We found a significant number of children demonstrating abnormalities as detected by neurologic (29%) and neuro-imaging examinations (44%). The majority of children with neuro-imaging abnormalities presented with ventricular enlargement and/or sulcal widening (29%) or white matter lesions (39%). Neurological abnormalities such as language impairment and developmental delay were frequently detected in HIV-1 infected children and were significantly associated with ventricular enlargement and/or sulcal widening.

Ventricular enlargement and/or sulcal widening were not associated with HIV-1 related markers or duration of cART. White matter lesions were associated with HIV-1 related parameters including blood and CSF HIV VL at time of baseline neuro-imaging. Duration of cART usage was not associated with presence of white matter lesions. Furthermore, in cART treated children, white matter lesions did not improve in time. Congenital CMV and toxoplasmosis infection that are associated with HIV-1 infections were detected in 2 children. These children did not have white matter lesions. Their neurologic examinations also remained stable over time.

Previous studies reported substantial survival benefit of different cART regimes after diagnosis of HIV-1 encephalopathy and improvement of impact neuro-imaging with CNS penetrating ART[1], [13], [22]–[24]. Duration of cART usage and specific PI- or NNRTI regimes did not impact the presence of ventricular enlargement and/or sulcal widening or white matter lesions in our study. The cART regimes used in our study are reported to have a moderate to good penetration of the CNS [25]. Associations with HIV-1 related parameters and lack of associations with cART treatment imply that the initial phase of active viral replication in CNS prior to cART treatment is crucial in the development of neuro-imaging abnormalities. Nevertheless, the small sub-study showed that ventricular enlargement and/or sulcal widening decreased in approximately half of cART children. These results must be interpreted cautiously but could indicate that long term cART can indeed reduce certain neuro-imaging abnormalities, while other specific neuro imaging abnormalities such as white matter lesions may remain irreversibly present over time. Rapid initiation of treatment may be helpful to prevent progression of white matter lesions and potentially reverse widening of the sulci to improve long-term neurologic outcome [16], [24]–[26]. Lack of significant associations of neuro-imaging abnormalities with cART may be due to the small sample size in our study. Nevertheless our results show that neuro-imaging abnormalities and neurologic abnormalities at examination are still frequently detected in the era of cART in the pediatric population and suggest that rapid initiation of treatment is critical to prevent progression of neurological abnormalities. Further prospective research in larger cohorts is required to assess the long-term effect of high CNS-penetrating regimens to improve the neurological outcome in HIV-1 infected children.
